# Design Research to Embed mHealth into a Community-Led Blood Pressure Management System in Uganda: Protocol for a Mixed Methods Study

**DOI:** 10.2196/46614

**Published:** 2023-11-30

**Authors:** Josephine Schwab, Jonas Wachinger, Richard Munana, Maxencia Nabiryo, Isaac Sekitoleko, Juliette Cazier, Rebecca Ingenhoff, Caterina Favaretti, Vasanthi Subramonia Pillai, Ivan Weswa, John Wafula, Julius Valentin Emmrich, Till Bärnighausen, Felix Knauf, Samuel Knauss, Christine K Nalwadda, Nikkil Sudharsanan, Robert Kalyesubula, Shannon A McMahon

**Affiliations:** 1 Heidelberg Institute of Global Health Heidelberg University Hospital Heidelberg Germany; 2 African Community Center for Social Sustainability Nakaseke District Uganda; 3 Department of Community Health and Behavioral Sciences School of Public Health, College of Health Sciences Makerere University Kampala Uganda; 4 mTOMADY gGmbh Berlin Germany; 5 Department of Nephrology and Medical Intensive Care Charité Universitätsmedizin Berlin Berlin Germany; 6 Professorship of Behavioral Science for Disease Prevention and Health Care, TUM School of Medicine and Health Technical University of Munich Munich Germany; 7 Department of Neurology with Experimental Neurology Charité Universitätsmedizin Berlin Berlin Germany; 8 Department of Internal Medicine School of Medicine Yale University New Haven, CT United States; 9 Department of Physiology College of Health Sciences Makerere University Kampala Uganda

**Keywords:** Uganda, hypertension, mobile health, mHealth, mobile money, pooled financing, medication availability, human-centered design, mixed methods, mobile phone

## Abstract

**Background:**

Uncontrolled hypertension is a leading risk factor for cardiovascular diseases. In Uganda, such diseases account for approximately 10% of all deaths, with 1 in 5 adults having hypertension (>90% of the hypertensive cases are uncontrolled). Although basic health care in the country is available free of cost at government facilities, regularly accessing medication to control hypertension is difficult because supply chain challenges impede availability. Clients therefore frequently suspend treatment or buy medication individually at private facilities or pharmacies (incurring significant costs). In recent years, mobile health (mHealth) interventions have shown increasing potential in addressing health system challenges in sub-Saharan Africa, but the acceptability, feasibility, and uptake conditions of mobile money approaches to chronic disease management remain understudied.

**Objective:**

This study aims to design and pilot-test a mobile money–based intervention to increase the availability of antihypertensive medication and lower clients’ out-of-pocket payments. We will build on existing local approaches and assess the acceptability, feasibility, and uptake of the designed intervention. Furthermore, rather than entering the study setting with a ready-made intervention, this research will place emphasis on gathering applied ethnographic insights early, which can then inform the parameters of the intervention prototype and concurrent trial.

**Methods:**

We will conduct a mixed methods study following a human-centered design approach. We will begin by conducting extensive qualitative research with a range of stakeholders (clients; health care providers; religious, cultural, and community leaders; academics; and policy makers at district and national levels) on their perceptions of hypertension management, money-saving systems, and mobile money in the context of health care. Our results will inform the design, iterative adaptation, and implementation of an mHealth-facilitated pooled financing intervention prototype. At study conclusion, the finalized prototype will be evaluated quantitatively via a randomized controlled trial.

**Results:**

As of August 2023, qualitative data collection, which started in November 2022, is ongoing, with data analysis of the first qualitative interviews underway to inform platform and implementation design. Recruitment for the quantitative part of this study began in August 2023.

**Conclusions:**

Our results aim to inform the ongoing discourse on novel and sustainable pathways to facilitate access to medication for the management of hypertension in resource-constrained settings.

**Trial Registration:**

German registry of clinical trials DRKS00030922; https://drks.de/search/en/trial/DRKS00030922

**International Registered Report Identifier (IRRID):**

DERR1-10.2196/46614

## Introduction

### Background

Globally, noncommunicable diseases (NCDs) are becoming the leading cause of death [1]. Further exacerbated by aging populations, NCD-associated mortality is expected to exceed infectious and respiratory causes of death by 2030 [2]. Cardiovascular diseases (CVDs) make up 13% of the mortality rate in sub-Saharan Africa (SSA) and account for the largest proportion (37%) of deaths caused by NCDs [3], adding to a double burden of communicable diseases and NCDs. Although the age-adjusted CVD mortality rates are lower in SSA than in high-income countries, the absolute number of CVD-related deaths in SSA has increased significantly in the past three decades, largely due to rapid urbanization, population growth, aging, dietary and lifestyle changes [4,5].

The successful management of CVDs requires controlling common risk factors (such as hypertension and diabetes) and ensuring that access to required medications is stable. The latter point is particularly challenging in settings marked by scarcity. In Uganda, an estimated 26.4% of adults have high blood pressure, and another estimated 36.9% of the country’s population are estimated to be prehypertensive (with a blood pressure reading of >120/80 mm Hg) [6]. Among those who are prehypertensive, approximately one third could become hypertensive within 4 years [7]. Although basic health care is meant to be available free of cost at government facilities across the country, the availability of essential medicines for conditions requiring long-term management, including hypertension, is generally low [8-10]. Clients who do not receive treatment at government facilities must buy prescribed medicines at private health facilities or pharmacies, where prices tend to be 3 to 4 times higher than at public facilities [11]. Out-of-pocket payments represent 39% of the total health care expenditures in Uganda [12], often exceeding the resources of many households and causing noncompliance with prescribed regimens [13-15]. The resulting regular disruptions in long-term medication schedules can result in uncontrolled blood pressure associated with a high CVD risk.

However, uncertainty remains in terms of potential interventions to sustainably address gaps in NCD care in SSA in general and Uganda in particular. Several novel approaches have been recently trialed, including community health worker (CHW) mobilization in South Africa [16] and medication adherence clubs in Kenya [17]. Other novel approaches have explored mobile health (mHealth) to target medication adherence (eg, SMS text message reminders for tuberculosis medication [18] or an electronic app for self-monitoring in Uganda [19]). Dialogue within our team (comprising anthropologists, epidemiologists, clinicians, nurses, community outreach workers, community leaders, and policy makers) therefore guided us toward mHealth solutions with a particular focus on mobile money, given that medication adherence is greatly affected by financial resources [20].

Mobile money, which refers to payments made via a digital wallet on a mobile device, has been evaluated in several studies for health interventions in SSA to address health system challenges in agile ways, including mobile money transfers to pay transportation costs for patients with cancer in Malawi [21] or to facilitate health insurance uptake in Kenya [22]. In Uganda, mobile money programs across the country have provided a savings mechanism to many people who were previously unable to access formal bank accounts; in 2021, approximately 71% of individuals were registered for a mobile money account [23] (compared with 29% of individuals having access to a formal bank account [24]). However, although a range of sectors have used mobile money approaches to facilitate payment for, among others, school fees, bills, water or food [25,26], application in the context of health interventions in Uganda has so far been limited.

To the best of our knowledge, no interventions have yet explored the potential of mobile money approaches in alleviating financial difficulties for hypertension treatment. Although the interest in mHealth interventions in general is rising, and the potential of mobile money for economic equality in particular is high [27], their application might be associated with implementation challenges, with scholars highlighting the complexity of mHealth interventions, particularly in relation to accessibility [28]. However, person-based development strategies have shown promising results in terms of increasing the interventions’ acceptability and feasibility [29,30], but these strategies remain underused when exploring the potential of mobile money approaches to chronic disease care. This study seeks to fill this gap in the literature by applying human-centered design (HCD) to co-design and test an mHealth blood pressure management intervention in rural Uganda, assessing the design process as well as intervention uptake and viability.

### Study Objectives

This study aims to codevelop (with end users and implementers) an integrated mobile money–based solution for antihypertensive medication delivery and to assess the platform’s feasibility, acceptability, and effectiveness.

The specific study objectives are as follows:

1. Understanding client, provider, and intervention implementer perspectives on financing schemes and mHealth interventions that are meant to increase medication availability and uptake among clients with NCDs (work package [WP] 1a).
2. Developing together with end users a prototype for a mobile phone–based solution to increase the availability and uptake of antihypertensive medication and assessing the development and design process (WP 1b and WP 2).
3. Testing the prototype and evaluating end users’ and health providers’ experiences with 2 versions of the prototype, including how users make and evaluate payment management decisions (WP 3) and whether 1 version is more effective than the other.

## Methods

### Study Setting

**Figure 1:**
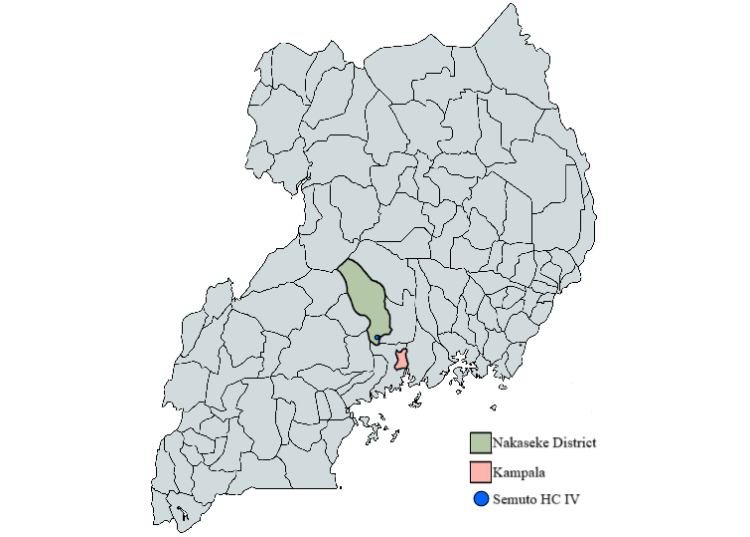
Map of Uganda highlighting the location of our study setting: Nakaseke district and Semuto Health Center (HC) IV.

This design research study will take place in rural Nakaseke district, Uganda, with a population of 29,400 [31] (Figure 1). Nakaseke administratively belongs to the central region of Uganda, where an estimated 28.5% of the population are hypertensive [6]. Our study will focus on clients within Semuto Health Center IV, chosen during an early formative phase of research because the center has initiated an alternative, promising approach to manage blood pressure, which is described in the following subsection.

### Semuto Health Center IV’s Alternative Approach: A Community-Led Pooling Strategy

Recognizing the challenges of medication supply, the clients and staff of a designated NCD treatment unit at Semuto Health Center IV collectively initiated a client self-help financing scheme wherein clients living with hypertension were invited to pay 2000 Ugandan shillings (US $0.54) per clinic visit, and clients living with diabetes were invited to pay 7000 Ugandan shillings (US $1.87) per clinic visit. These funds were then pooled to purchase medication. This initiative sought to increase medication availability by buying in bulk via a wholesale vendor, which is a more cost-effective approach than purchasing medication from a retail vendor. Payment was voluntary; those who contributed to the pool could draw on the additional allotment, whereas those who did not contribute could access government-supplied medication. Beyond purchasing blood pressure medication, excess funds were also used to purchase equipment such as batteries or glucose strips. The scheme, although promising, was not reliably generating sufficient funds to ensure a continued supply of medications and proved challenging in terms of paperwork, accounting, and management. A nurse was assigned the task of purchasing medication from a central pharmacy in the capital, Kampala, but real-time knowledge regarding the amount of funding available complicated her purchasing trips. Furthermore, the clinic staff struggled to track who had made or not made payments, which would affect who could draw from the specially purchased pool of medication.

### Semuto Health Center IV Approach 2.0: The MoPuleesa Intervention

**Figure 2:**
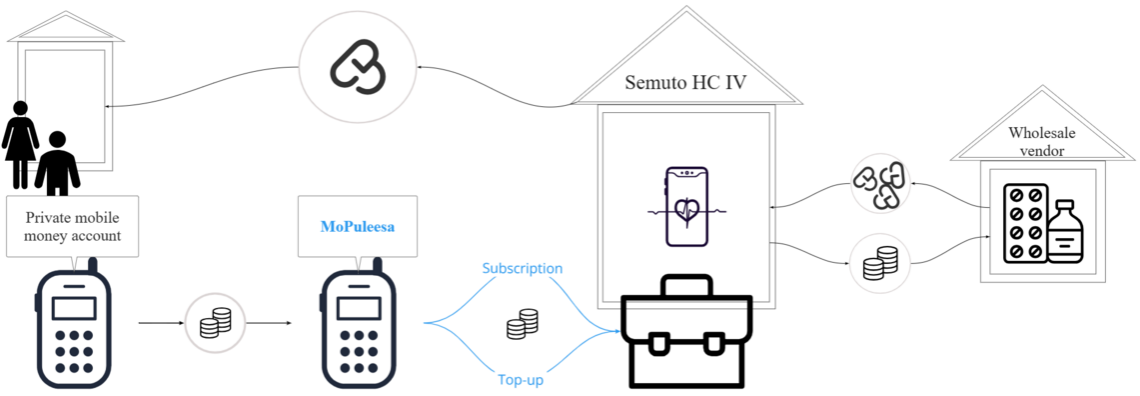
Flow of money from the client via their mobile health wallet to the Health Center (HC) and medication flow back to the client.

Building on the paper-based approach of Semuto Health Center IV, we will digitize and expand the existing system, drawing on insights from community members, software programmers, anthropologists, social scientists, health care providers (HCPs), mHealth implementers, policy makers, and local leaders with an aim to create a continual flow of money to secure medication availability. The proposed money flow is illustrated in Figure 2.

The central component of the co-designed intervention will be the mHealth wallet (illustrated as the *MoPuleesa feature phone* in Figure 2 [*puleesa* means “pressure” in the Luganda language]), which will allow clients to deposit the money they wish to save for their health care expenses via their private mobile money accounts. Others (eg, family members) who are themselves not users of the system can also remotely contribute to a client’s mHealth wallet. Clients will be prompted to transfer money from their account to the clinic pool to facilitate bulk medication purchasing via 1 of 2 ways: either allowing for a specific amount of money to be automatically transferred from a marked savings account via direct deposit at predetermined and fixed intervals (the *subscription approach*) or independently transferring amounts of their own choosing at any time within a 1-month period (the *top-up approach*), shown by the corresponding arrows in Figure 2. Clients using the subscription approach who want to change the subscription amount or frequency can approach the designated clinic staff in person at the health center. For the top-up approach, clients can choose the timing themselves but can only contribute the exact amount needed to avail drug coverage each month; excess contributions will not be transferred to the following months. Core principles guiding our design and implementation efforts are the engagement of CHWs for community commitment, the use of mHealth technologies not requiring costly infrastructure at an individual level, and the integration of the designed intervention into existing clinic structures via a multidisciplinary team.

To facilitate end-user buy-in, we will work with CHWs, who are volunteers serving as intermediaries and communication links between clients and medical facilities within their own communities, which often imbues them with considerable trust, experience, and communicational skills [32,33]. In our study, we will include CHWs not only for outreach and recruitment purposes but also as key informants who can provide valuable insights on setting and intervention design, supporting a sustainable outreach that in turn might enhance the engagement of the community with the intervention and assist regular contributions.

For the integration of a novel technology, we acknowledge the need for easy accessibility by all members of the community; therefore, the platform does not rely on users having their own smartphone or an internet connection. Instead, following successful examples [34,35], all system functions will be accessible from feature phones via unstructured supplementary service data (USSD) menus, which operate via service and control commands in a mobile network. To access the USSD menu, users will be able to either register their own mobile phone number or receive a new SIM card in their name upon study enrollment (with which they can access their personal account via the mobile phones of CHWs, other clients, or any friend or family member). For validation purposes, the study team will routinely conduct on-site user research with stakeholders and end users to ensure that the envisioned flows and developed prototypes fit the needs of end users. Following the tenets of HCD, this validation process is directly integrated into the prototype codevelopment and product design.

### Theory of Change

**Figure 3:**
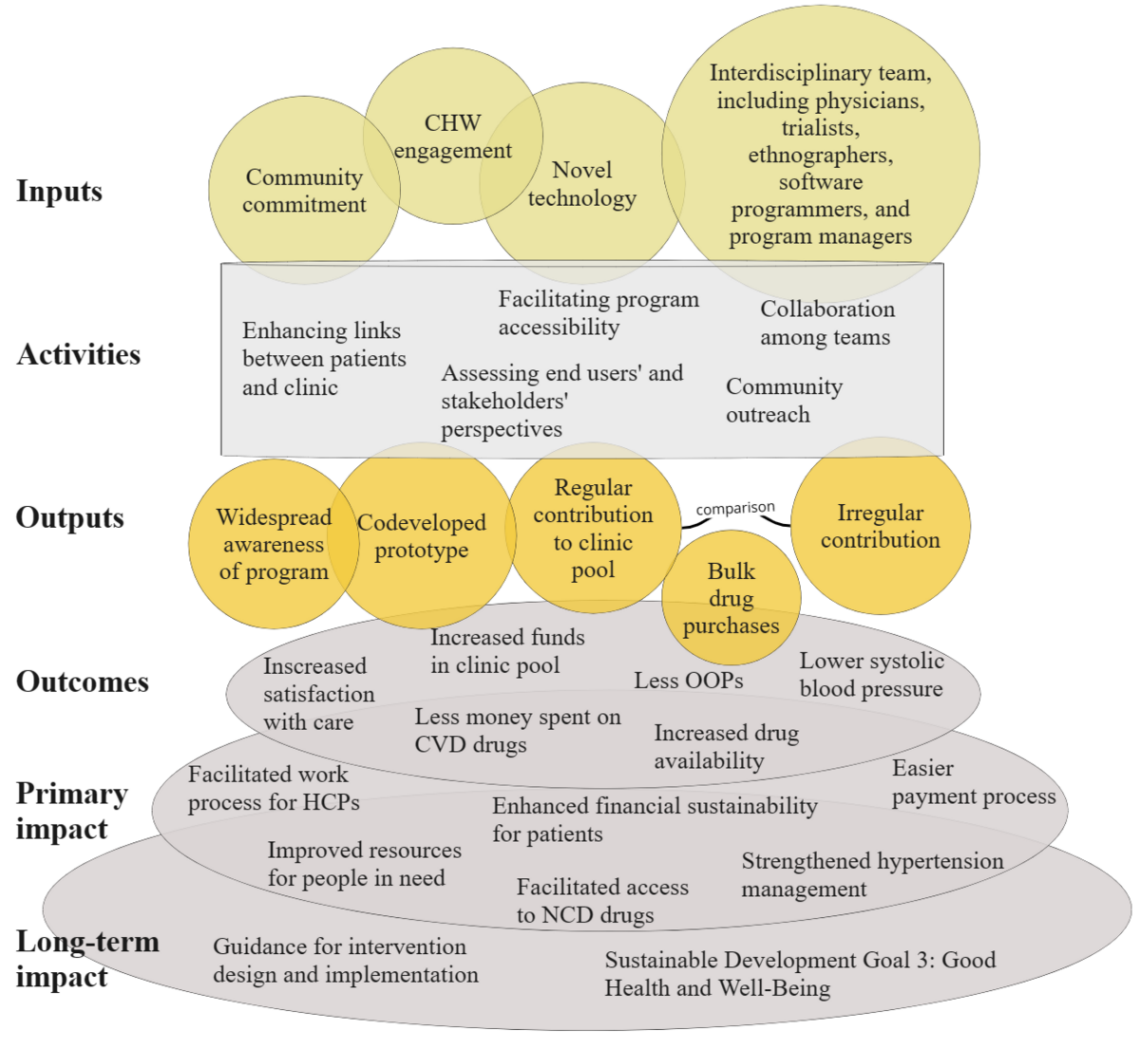
Theory of change illustrating inputs and activities, with expected outputs, outcomes, and impact. CHW: community health worker; CVD: cardiovascular disease; HCP: health care provider; NCD: noncommunicable disease; OOP: out-of-pocket payment.

Building on the envisioned MoPuleesa intervention approach, Figure 3 summarizes key inputs, activities, and causal pathways that underpin our design and implementation work, serving as a guide to understand how the intervention will lead to the desired outcomes and impact.

We theorize that the mHealth platform will foster a regular influx of money into the pooled system, which will allow the clinic to purchase antihypertensive medication in bulk via designated channels (medication purchase by the health center directly from wholesale vendors shown in Figure 2), thereby reducing costs for individual clients (compared with out-of-pocket purchasing of medication at pharmacies) and facilitating a sustainable, stable supply of medication. Considering a possible scale-up of this intervention and learning from our design and implementation process, we envision a long-term impact (shown in the increasingly larger circles in the lower part of Figure 3) on the well-being of patients with hypertension and on scientific intervention design efforts. Our envisioned methodological approaches to achieve this goal and the overarching study objectives are outlined in detail in the following subsections.

### Methodology

Originating in computer science, artificial intelligence, and ergonomics, HCD has more recently begun to inform how public health actors design and implement health interventions [36]. In the HCD approach, end users are involved in all stages of the design process and have influence on the final product of the intervention, with the implicit rationale that engaging users facilitates implementation success [37]. HCD begins with developing a thorough understanding of the status quo in question to define an issue that will be addressed. On the basis of this understanding, researchers and end users together develop an idea for a potential product or intervention (empathize, define, and ideate). As this process continues, researchers elicit feedback to iteratively adapt the envisioned solution to end users’ needs and expectations to maximize the fit of the developed prototype to the setting at hand (prototype). Finally, the developed product is pilot-tested (test).

As a design-driven study, this research did not begin with a predetermined intervention in mind; instead, we sought to capture the nature of the challenge and potential solutions, identified as promising, in similar settings. We use the HCD approach to identify product priorities and intervention approaches and to codevelop product prototypes together with end users. Once a final product is identified, it will be evaluated quantitatively for efficacy, usability, and desirability.

To provide maximum experience and expertise, we will leverage the multidisciplinarity of our team throughout the project with a focus on trust, communication, and team identification within the team to facilitate collaborative work [38]. Our team includes ethnographers and social scientists who will ensure context sensitivity and qualitative insight into end users’ and stakeholders’ experiences, preferences, and perspectives; local clinical research leaders who will tap into context expertise and communication channels; medical professionals, including biomedically trained clinicians, who will assess the medical aspects and health benefits of the intervention and gain insight into the local health system; NCD-focused trialists who can meaningfully incorporate quantitative results that can guide further research directions; and, finally, software programmers and experts in the technical aspects of the project who will guide successful implementation, identify opportunities, and make the intervention as up to date and innovative as possible.

### Research Design

**Figure 4:**
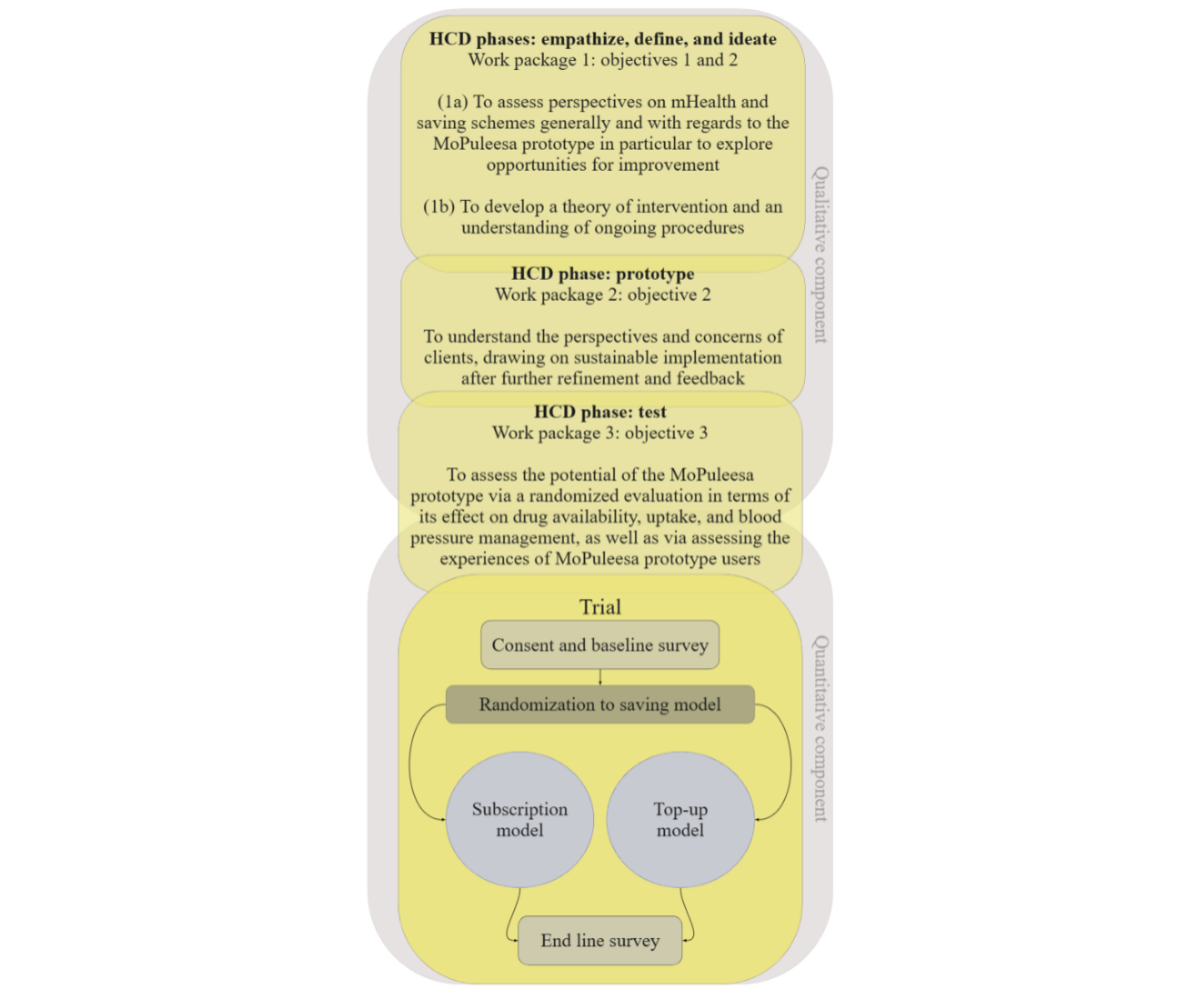
Summary of all study phases and methods (including trial details) corresponding to specific study objectives. HCD: human-centered design; mHealth: mobile health.

This study will follow a mixed methods exploratory sequential design [39] in line with established recommendations for HCD studies [40,41]. In a first step, the design approach will allow us to qualitatively explore stakeholder perceptions, experiences, and recommendations in the setting at hand, informing key design decisions in accordance with specific study objectives 1 and 2. Although formative research revealed the essential challenge of accessing drugs in the study setting and recognized mobile money as a potential solution, qualitative data are needed to inform the design and operationalization of the platform, anticipate potential difficulties and facilitators, and assess clients’ willingness to accept the approach. Building on these qualitative results, while maintaining core trial design and rationale, we will refine and finalize the concluding quantitative component aimed at pilot-testing the intervention in the form of a randomized controlled trial (study objective 3). Sequentially combined, these methods will allow us to ideate and design an intervention adapted to the specific study context, as well as allow for evidence-based conclusions regarding the prototype’s potential in facilitating sustainable hypertension care. Figure 4 highlights the overlap of research activities and WPs across the HCD phases.

### HCD Phases

#### Empathize, Define, and Ideate: WP 1

WP 1 is divided into 2 phases, both informed by the tenets of HCD. In the first phase (WP 1a), we will conduct in-depth interviews (IDIs) and focus group discussions (FGDs). The IDIs will facilitate the capture of clients’ personal perspectives on, and experiences with, blood pressure management and hypertension, mHealth, savings schemes, and the existing paper-based scheme that inspired this work. We will explicitly probe on more nuanced facets such as trust and transparency. In addition to IDIs, we will conduct FGDs to determine how groups such as HCPs, clients, and CHWs negotiate the trade-offs and opportunities of different approaches to hypertension management and care, including the proposed intervention. Within the FGDs, the envisioned platform use process will be explained to participants step by step, and feedback will be gathered. We will collectively determine intervention facets, such as how the intervention should be explained or presented within a community, who should be responsible for enrolling clients and troubleshooting challenges, what foreseeable challenges respondents see with the intervention, and how these should be circumnavigated or addressed. Finally, within WP 1a, we will examine field notes from the previous phases of formative research, where we collected information on CVD management in Nakaseke region from NCD clients and HCPs; these notes will bolster our own recall of the system in place at Semuto Health Center IV and its development, which can inform how we can build a meaningful product.

In a second phase (WP 1b), we will conduct nonparticipant semistructured observations of client-provider interactions and clinic processes, as well as IDIs with higher-level stakeholders. The observations will be conducted by research team members working in Nakaseke district who will already have interacted with clients and providers on site; this will allow us to understand actual behaviors and current workflows regarding hypertension management and care in the study setting (including, eg, how client-provider interactions and medication procurement or delivery currently take place). Using an observation checklist, we will examine how health services related to blood pressure management are currently provided and how clients are handled at Semuto Health Center IV. In addition, we will gather information on the locations and processes of medication prescription, storage, and delivery. In the stakeholder IDIs, we will capture insights from policy makers, academics, and public health actors at community and national levels to determine the feasibility, opportunities, and potential pitfalls of our proposed intervention, both with regard to our target setting and when considering how to scale-up our intervention. Table 1 summarizes the individual activities conducted in WP 1a and WP 1b.

**Table 1:** Methods used in work package (WP) 1: in-depth interviews, focus group discussions, and document analysis.

Activities			Participants		Themes		Purpose
**WP 1a**							
	IDIs^a^ (n=20-40)	Clients visiting the NCD^b^ clinic at Semuto Health Center IV		Hypertension management and care Mobile health Money-saving schemes Feedback on early concepts for the intervention		To identify essential facets to include in the prototype	
	FGDs^c^ (n=5)	Clients and CHWs^d^		Perspectives on planned intervention Communities’ hypertension experiences and perspectives		To identify possible facilitators and barriers to intervention implementation	
	Document analysis	Field notes		Field notes from formative research phase		To gather information on what has informed the existing approaches and identify key stakeholders	
**WP 1b**							
	Semistructured observations (n=3-5 clinic days)	HCPs^e^, clients, and staff at the health center		Client-provider interaction (n=10) Clinic processes Medical examination Medication procurement and delivery Locations of medication supplies, output, or storage		To understand current workflows regarding NCD management and care	
	IDIs (n=10-20)	Academics, implementers working on CVDs^f^ or mHealth^g^ interventions, and policy makers		Development and implementation of mHealth interventions Research on NCD management Current health care services		To develop a theory of intervention for implementation and scale-up	

^a^IDI: in-depth interview.

^b^NCD: noncommunicable disease.

^c^FGD: focus group discussion.

^d^CHW: community health worker.

^e^HCP: health care provider.

^f^CVD: cardiovascular disease.

^g^mHealth: mobile health.

#### Prototype: WP 2

WP 2 (Table 2) aims at finalizing the design prototype and preparing for pilot testing. To collect detailed feedback from end users and ensure that the final product aligns with their expectations, we will present a first MoPuleesa prototype on mobile devices to the end users. Luganda-speaking research team members on site will conduct think-aloud exercises, which will allow a much more targeted refinement of the prototype to address emerging barriers or challenges [42]. Think-aloud exercises are used to track a participant’s thoughts while using a platform and to capture their problem-solving process [43]. After a short practice phase, participants are asked to comment on each step they take while using the platform and to say out loud what thoughts come to mind [42]. To minimize potential biases, data collectors will be asked to not interrupt the exercise and not interact with participants during the exercise unless they stop speaking (whereupon participants will be encouraged to continue speaking). Furthermore, we will capture local policy makers’ and local leaders’ feedback on the prototype and envisioned implementation processes via IDIs to further refine the prototype and identify potential difficulties and facilitators for rollout.

**Table 2:** Methods used in work package 2: think-aloud exercises and in-depth interviews.

Activities	Participants	Themes	Purpose
Think-aloud exercises (n≤40)	Clients, HCPs^a^, CHWs^b^ (who have or have not used a version of the prototype before)	Interaction with latest prototyped platform Usability of platform features	To refine the prototype to address emerging barriers or challenges
IDIs^c^ (n≤20)	Key stakeholders (policy makers and local leaders)	Latest prototype version Benefits and barriers Implementation process	To incorporate further concerns and perspectives

^a^HCP: health care provider.

^b^CHW: community health worker.

^c^IDI: in-depth interview.

#### Test: WP 3

In WP 3 (Table 3), we will pilot-test the final prototype. For the qualitative component of this WP, we will conduct IDIs with clients who were particularly engaged in previous interviews or who suggest during trial administration that they would like to expand on their responses in more detail to get a full understanding of their perspectives on the design and implementation process. In addition, to provide guidance on best practice approaches to co-designing mHealth interventions with end users, we will conduct IDIs with key stakeholders involved in this process, including health center staff, software developers, and members of the study team.

Quantitatively, we will assess differences between the top-up and subscription contribution options, as well as the impact of the intervention on service use, user satisfaction, intervention uptake and funds contributed, and clients’ blood pressure readings, in a 2-arm between-participants parallel randomized controlled trial.

Participants will be randomized to 1 of the following 2 intervention arms (for the randomization plan, refer to the *Sample, Sampling, and Data Collection* subsection):

**Table 3:** Methods used in work package 3: in-depth interviews and randomized controlled trial.

Exercise			Participants		Themes		Purpose
**Qualitative**							
	IDIs^a^ (n=5-7)	Clients (particularly engaged in previous interviews or suggested during survey administration that they would like to expand on their responses in more detail)		Expectations toward the prototype Perception of implementation process		To assess experiences with the finalized prototype	
	IDIs (n=10-15)	Key stakeholders involved in the implementation process (health center staff, software developers, and members of the study team)		Team communication Implementation process Problem-solving		To provide guidance on best practice approaches to co-designing mHealth^b^ interventions with end users	
**Quantitative**							
	Trial (sample size: 250-350 clients)	All clients who have given consent to participate in the trial and take up a money-saving model		Baseline and follow-up survey Kano questionnaire [44] Aggregation of clinic data		To gather information on trial participants	

^a^IDI: in-depth interview.

^b^mHealth: mobile health.

Arm 1: subscription-based model, where individuals will be given the opportunity to enroll in a medication-specific mobile money savings account where they can deposit money over the course of the month. If clients reach the minimum amount needed to purchase medications for the following visit, the money will be automatically debited from their savings account at the end of each month.

Arm 2: top-up payment model, where clients can actively make mobile money transfers to the health facility throughout the month for the purchase of medication in the subsequent month without the option to make marked contributions for several months in advance.

### Main Hypothesis

For the comparison between these 2 subscription approaches (specifically study objective 3), we draw on ongoing discourses in the literature regarding the chances and challenges of monetary contribution approaches for various purposes. Our considerations included findings regarding the differences between restrained commitment accounts and flexible liquid accounts that suggest greater willingness to save with less account flexibility in cases where clients self-report awareness of their own limited saving discipline [45]. In addition, several scholars have suggested that marked savings accounts, similar to our proposed subscription-based model where clients can save money to cover automatically deducted monthly medication costs, increase utility costs for the later repurposing of already made marked savings and thereby can increase overall savings made for the intended purpose [46,47]. Furthermore, Fisher and Anong [48] have found that long-term saving motives as well as low risk tolerance might add to regular savings. Transferred to the setting at hand, this suggests that marked savings combined with regular and automated contributions might be preferred to ensure sustainable contribution and treatment.

When testing the developed prototype, our main hypothesis therefore is that the subscription model, combining benefits from committed, semiliquid, and long-term savings possibilities, will lead to more clinic visits, more intervention take up, and greater funds contributed to the health facility for the purpose of purchasing essential medications. We argue that, thanks to the ability to save dedicated funds in small increments for the explicit purpose of accessing a guaranteed medication stock, individuals in the subscription arm will be more likely to contribute to, and pick up, antihypertensive medications than individuals in the top-up payment arm. We will test whether this will lead to monetary savings (eg, individuals receive their medications through the public facility at a lower total cost than when purchasing out of pocket from a private pharmacy) and improved health (eg, individuals will be more consistent with picking up their medication and thus have improved blood pressure). We will evaluate the effect of the subscription model compared with that of the top-up payment model based on the outcomes listed in Table 4.

**Table 4:** Outcomes.

Category	Outcome	Measurement
Primary outcome	Number of NCD^a^ clinic visits as well as number of medication pickups	Health facility records
Implementation and medication outcomes	Percentage of people who take up funding models Total amount contributed to the clinic funds	Directly from MoPuleesa platform
Long-term health outcome	Systolic and diastolic blood pressure readings	Collected through CHWs^b^ and health facility records
User experience outcome	User satisfaction	Questions on user satisfaction in baseline and follow-up surveys

^a^NCD: noncommunicable disease.

^b^CHW: community health worker.

Participants will be asked to fill out a questionnaire at baseline and after 6 months that will address care-seeking behavior, the consistency of medication pickups, health-related money-saving routines, and the perceptions of care quality. At baseline, the questionnaire will also include sociodemographic characteristics. At 6 months, the questionnaire will further include questions about the feasibility and acceptability of the intervention, as well as a short series of platform attribute or Kano-style questions (the Kano model describes the relationship between product attributes and user satisfaction or disapproval to determine the relative importance of product features [44]). In addition, we will use data routinely collected at clinic level (demographic data, blood pressure readings, and height and weight measurements) and via the MoPuleesa prototype (money contributed and medication ordered) as detailed in Table 4.

### Participants and Inclusion Criteria

Across all WPs, the respondent groups include HCPs at facility and community levels (medical doctors and clinic staff), CHWs working in the community, community members and key stakeholders (religious and local leaders, members of pooled financing schemes, and academics), clients (diagnosed with hypertension and living within the catchment area of Semuto Health Center IV), and decision makers (policy makers and ministry of health representatives). In the final WP, we also include study team members involved in intervention design, implementation, and evaluation processes as respondents.

Ethnicity, race, political orientation, religion, and class are not criteria for inclusion or exclusion in this study. Respondents must be aged ≥18 years to participate. Persons who are incapacitated (mentally challenged or physical disability: hearing and speech impaired) are excluded from this study. Textbox 1 summarizes the inclusion and exclusion criteria for this study.

**Study inclusion and exclusion criteria.**

**Inclusion criteria (aged ≥18 y and having given informed consent)**

- Health care providers: community health workers, medical doctors, clinic staff working at Semuto Health Center IV or in community outreach in Nakaseke district, etc
- Local key stakeholders: religious and local leaders, members of financing schemes, academics, etc
- Clients: clients who have been diagnosed with hypertension and are registered at Semuto Health Center IV
- Decision makers: policy makers, ministry of health representatives, health insurance representatives, district administrators, etc
- Study team members: individuals involved in the intervention design, implementation, and evaluation

**Exclusion criteria**

- Refusing or withdrawing informed consent
- Not responding to invitations to participate
- Persons who are incapacitated (mentally challenged or physical disability: hearing and speech impaired)

### Sample, Sampling, and Data Collection

#### Qualitative Components

For the qualitative components of this study, we expect to recruit a maximum of 160 individuals. In line with the tenets of saturation (new data cease to provide fundamentally novel insights) [49] and information power [50], our sample size will be determined as data are collected. The aforementioned estimate is based on experience and an effort to achieve a justifiable trade-off between collecting high-quality data and minimizing strain on the target populations (eg, individuals to be interviewed) [51].

We will use purposive sampling [52] for WP 1 and WP 2 to gain a range of perspectives. Local leaders, policy makers, and academics will be contacted and recruited through the official communication channels for the respective health facility or office or via the established channels of African Community Center for Social Sustainability (ACCESS) Uganda. Health care professionals will be contacted through the respective clinics or when conducting structured observations once the clinics have agreed to participate. We will use snowball sampling [53] as necessary to contact additional participants.

For clients, convenience sampling [52] will be used. Clients will be approached during structured observations in the clinic areas where they await NCD care (waiting time: minimum 30 min). As clinic days for NCD care are conducted once a week at the health facility, clients will additionally be purposively recruited via local HCPs guiding the study team toward a range of clients who represent different types of background characteristics using the sampling criteria: age group (those aged >50 y and those aged <50 y), gender (male and female), and condition (hypertension, diabetes, or both). The study will be briefly introduced using an information sheet. If clients signal interest to participate, contact information will be exchanged to schedule an IDI. All IDIs and FGDs will take approximately 60 minutes and will be conducted by researchers and research assistants, including CHWs who work in the area but not in the study setting. All research assistants will have gone through a 5-day training program in qualitative research and interviewing and will work together as teams of 2 people representing a moderator and a notetaker. Research assistants will be provided with interview guides that will be refined in group sessions during the 5-day training period and pilot-tested on 2 days with participants within the study setting.

#### Quantitative Component

For the quantitative component, we anticipate enrolling between 200 and 350 clients. Client recruitment will follow the same procedures as those outlined in the previous subsection. This number was not the result of a detailed power calculation; rather, it was determined and constrained by the total client population from which we can recruit. Specifically, based on our discussion with the administrators of the NCD study clinic at Semuto Health Center IV, the clinic currently serves approximately 380 clients who meet the aforementioned inclusion criteria. Thus, we realistically expect to enroll 200 to 350 clients based on exact attendance during the study period and refusal rates.

#### Sample Size Estimation

On the basis of the range of recruited participants, we estimate the following minimal detectable effects (Table 5). For these calculations, our primary outcome is the number of clinic visits for medication pickups. On the basis of the 6-month study duration, this can range from 0 to 5 visits (individuals are asked to come once a month), which we will analyze as a count variable using Poisson regression. We additionally set an α of 5% and a power of 80%, with a 2-tailed *t* test for all estimates, and show the minimal detectable effects across a range of possible control counts (the number of visits for pickups among the voluntary contribution group in a range from 1 to 5) and sample sizes (ranging between 200 and 350). The minimum detectable effect is expressed as a rate ratio and measures the percentage increase in mean visits for the subscription contribution (intervention 1) compared with those for the voluntary contribution (intervention 2) over the 6-month study period.

**Table 5:** Minimum detectable effect size across sample sizes and base rates.

Mean number of visits in the voluntary contribution (intervention 2) group	Minimum detectable effect size			
	200^a^	250^a^	300^a^	350^a^
1	1.44	1.39	1.35	1.32
2	1.30	1.27	1.24	1.22
3	1.24	1.22	1.20	1.18
4	1.21	1.19	1.17	1.16
5	1.19	1.16	1.15	1.14

^a^Total sample size (50% intervention 1 and 50% intervention 2).

#### Randomization

Participants will be randomized to be offered either the subscription model or the top-up contribution payment model and assigned to the different models by study team members after the initial recruitment at the facility and upon providing informed consent. The allocation sequence for the random assignment of the intervention was developed by an independent statistician. Individuals are randomly assigned in a 1:1 ratio. We use stratified randomization by sex and day of recruitment, with participants randomized in blocks of 4 to maximize balance between treatment and control. The allocation is concealed because assignment is based on a random draw from a box such that an individual’s assignment is only revealed after they physically draw a slip.

#### Randomization Implementation

The quantitative research team developed the allocation sequence, and the research assistants enroll participants into the study. After enrollment into the study, each participant will be assigned to a particular arm by drawing concealed slips labeled either direct payment or savings from a box on the day of their recruitment into the study.

#### Blinding

The allocation sequence of assigning the intervention will be concealed from the research team until the moment of assignment. This will prevent selection bias when assigning participants to study arms. However, because of the nature of this intervention, blinding of neither the research team nor the participants will be possible once the program has started.

#### Data Management

The research study data manager will guide the data management responsibilities for this study. This study’s quantitative data will be collected using both paper and electronic case report forms, whereas qualitative data will be collected using audio recorders. Transcripts and field notes from the qualitative work as well as the paper case report forms will be kept under lock and key and will be overseen by the principal investigators. The transfer of participant-related data, if any, will be carried out in pseudonymized form; third parties will not have access to original data. REDCap (Research Electronic Data Capture; Vanderbilt University) will be used to enter, store, and manage quantitative data. To ensure that quality data are collected, the REDCap platform will provide an interface for data entry (an audit trail to track all changes within the database). In addition, automated validation checks will be embedded within the study database. The study data manager will share data queries generated from the accruing data with the research team on a biweekly basis.

### Data Analysis

#### Qualitative Data Analysis

Data generated from the qualitative interviews will be transcribed verbatim and analyzed using NVivo software version 14 (Lumivero) following a five-step framework approach: (1) *familiarization with the data*, (2) *identifying a thematic framework*, (3) *indexing*, (4) *charting*, and (5) *mapping and interpretation* [54]. This will help us generate an understanding of stakeholders’ experiences with the prototype. After the completion of the intervention prototype, a detailed analysis plan for this study will be developed. To adapt and inform our design and implementation processes, we will conduct systematic debriefings after each day of qualitative data collection. In weekly team meetings, we will share the first findings from these debriefings with all team members to adjust platform features to end users’ needs and facilitate implementation steps within the community.

#### Quantitative Data Analysis

We will summarize participant demographic characteristics and assess baseline balance between those randomized to the top-up and subscription arms. We will then assess the effect of offering the subscription model on the number of clinic visits with medication pickups (the main outcome) by comparing differences between those *offered* (including those who were offered and did not take up) the subscription model and those *offered* the top-up contribution model. As not all individuals will take up the offer, this will represent the intention-to-treat effect. We will estimate this effect using Poisson regression models. Next, we will assess the effect of the amount of money an individual contributed on the number of visits they attend. We will do this using a Poisson instrumental variable regression approach, where we use the random assignment as an instrument for the amount of money contributed. Finally, we will assess the effect of the random assignment to the different contribution arms on the proportion of individuals who take up the contribution model (the implementation outcome) using linear probability models and the effect on systolic and diastolic blood pressure readings using linear regression models.

#### Statistical Analysis

The baseline characteristics of participants will be compared between the 2 arms using medians and IQRs for continuous variables and proportions for categorical variables. Outcomes will be analyzed by both the intention-to-treat and per-protocol analysis methods using mixed effects Poisson regression methods for the primary outcomes. We will adjust for baseline imbalances for both the primary and secondary analyses, and both unadjusted and adjusted analyses will be presented. All our regression models will additionally control for baseline patient characteristics such as age, sex, and income to increase the precision of the treatment effects. We will not only conduct a complete case analysis but also present the results using multiple imputation as sensitivity analyses. Missing data on outcomes and key covariates will be assessed before analyses. Where applicable (missingness >10%), we will implement multiple imputation methods appropriate for random effects models. Two-tailed *P* values of ≤.05 will be considered to indicate statistical significance. Analysis will be performed with Stata software (version 17; StataCorp LLC). We will not conduct any interim analysis for this study.

### Ethical Considerations

The study protocol was approved by the institutional review board of the Medical Faculty, Heidelberg University, Heidelberg, Germany (S-299/2022) and the Makerere University School of Biomedical Sciences Research and Ethics Committee (SBS-2022-175), Kampala, Uganda. The study has been registered at the Uganda National Council for Science and Technology (HS2445ES). Protocol modifications will be communicated to both ethics committees, the Uganda National Council for Science and Technology, and the German Clinical Trials Register. All participants will provide written informed consent before their enrollment in the study. Consent will be obtained by the study research team.

### Dissemination of Findings

We will disseminate the findings from all study phases via established academic channels (eg, peer-reviewed publications and conference contributions). Furthermore, we will hold dissemination workshops with local stakeholders and policy makers to ensure transparent communication; to discuss the impact the intervention has had on local medication availability, purchase, and distribution; and, in the case of proof of concept, to explore the potential for scaling up and replicating our intervention.

## Results

This study was granted funding by the German Federal Ministry of Education and Research via the German Alliance for Global Health Research in April 2021 for a duration of 24 months, with an extension of 8 months until December 2023. The funders have no role in study design or analysis. As of August 2023, the first part of qualitative data collection had been finalized (with 55 IDIs and 4 FGDs conducted with clients, HCPs, CHWs, local leaders, and policy makers), and qualitative data analysis is underway. The results from the ongoing qualitative phase have informed platform design and implementation in accordance with the HCD principles outlined previously. In addition, the results are providing insights into current practices and understandings in the target population regarding topics such as NCDs, community-led health financing, and mHealth approaches. The findings are expected to be published in early 2024.

The randomized controlled trial for pilot-testing the final intervention prototype was registered in December 2022, paving the way for recruitment, which started in August 2023 and is ongoing. The results from the quantitative trial are expected to be published in summer 2024.

## Discussion

### Expected Observations

Hypertension is a key risk factor for CVDs and significantly contributes to morbidity and mortality across the globe, but accessing the required long-term care remains challenging, especially in low-resource settings. In this study protocol, we outlined how we will work with clients, providers, and other key stakeholders in a rural Ugandan setting to build on a community-led self-help financing scheme for facilitating access to antihypertensive medication. Following the tenets of HCD, we will rapidly iterate and refine an mHealth platform to incorporate end-user feedback across all stages of the design process.

Efforts to bolster sustainability and fairness in access to health care have a long history in global health. The large-scale multisectoral declaration of Alma Ata in 1978, aiming at adequate health care provision for everyone by 2000, has been criticized for the lack of follow-up on implemented goals and its selectiveness in the addressed diseases [55]. Placing a larger emphasis on the principle of strengthening primary health care, the Bamako Initiative of 1987 aimed at the integration of communities in attempts to facilitate health care financing. However, this initiative is widely considered to have failed, and it has been criticized for its top-down approach or in some instances increasing financial burden on some of the populations classified as the most disadvantaged [56]. In the aftermath of these large-scale efforts, researchers and implementers have highlighted still-open questions regarding pathways to avoid selectiveness in primary health care, to prevent shifts toward the private sector when client charges are introduced, and to meaningfully engage communities in implementation and follow-up [57]. In our study, we will contribute to providing answers to these questions, combining qualitative and quantitative approaches in the HCD design to gain insights into the perspectives and experiences of local stakeholders and end users.

In response to the lessons learned in the context of the previous efforts highlighted in this paper, we aim to build on a system initiated locally with already existing stakeholder buy-in, rather than implementing a new intervention created outside the study setting. We focus on a bottom-up approach to work with local realities and learn from clients’ and providers’ past experiences, and we have conducted extensive formative research in the study setting to inform the research and implementation activities presented in this protocol. The results of this study may inform future research on the potential of design research as a means to build on locally sourced approaches with high existing stakeholder buy-in.

### Addressing Reported Challenges

mHealth approaches in resource-constrained settings are often accompanied by many difficulties that could hinder their acceptance and uptake (eg, incomplete mobile phone access owing to lack of a personal mobile phone, charging challenges, or theft) [58]. Some studies report difficulties for mHealth interventions that rely on a stable network connection or complex digital software [28,59,60]. To facilitate accessibility and overcome the challenges reported by fellow researchers, the MoPuleesa platform is based on a simple USSD menu that can be operated from any feature phone without internet access. It is also linked to a SIM card that can be accessed via different devices and can be locked in case of theft. This way, we hope to contribute to the potential of digital technologies for health care to reach resource-limited areas. Furthermore, research has shown that trust and risk assessment play a crucial part in enhancing the uptake of mHealth interventions and mobile money acceptance [61]. Moreover, service quality and confirmed expectations in turn seem to increase confidence in an intervention [62]. We want to investigate these components within our quantitative evaluation to provide thorough feedback on the variables for intervention uptake and use.

Community financing approaches should consider mandatory contributions to facilitate sustainability [63]. However, end users and implementers in the context of our formative research have voiced concerns that in our setting, mandatory contributions might result in a decrease of community engagement and continual intervention uptake. Similar findings have been reported from research conducted in Nigeria [64]. As health care should be available free of cost at government facilities in Uganda, mandatory contributions might be perceived as unfair and an additional, often prohibitive burden. Instead, formative research in our setting has shown that a financing scheme led by the community can spark substantially more trust and willingness to participate if it is an optional addition to governmental health coverage. Despite the potential of such community-driven efforts, a tension remains between creating a program that is flexible in terms of contribution (as desired by clients who are contending with seasonal harvests and uneven employment) and building a program that is rigid in its contribution requirements (as desired by health providers who are trying to forecast bulk purchasing needs). To strike a balance between maintaining acceptability of the community-initiated financing program and facilitating a sustainable influx of funds, our comparators for pilot-testing the developed intervention will be based on 2 voluntary contribution approaches that differ in their rigidity.

To further facilitate a sustainable influx of money, previous studies have highlighted the importance of affordability and the amount of trust placed in the operating administrators [65]. In addition, both previous studies and our formative research have found that the acceptability of financing schemes might increase if contributors are closely involved in the creation and the management of a scheme [66]. Therefore, in our study, we aim to bolster trust by engaging CHWs, HCPs, and other local stakeholders throughout intervention design and rollout, and suggested contribution amounts will be determined based on conversations with both clients and providers.

Finally, the success of pooled financing approaches strongly depends on the number of regular contributors [67]. After proof of concept of our intervention, 1 key challenge to achieve long-term sustainability will therefore be to scale platform integration and participation to maintain the constant influx of money required for bulk medication purchasing. To explore options for scale-up at clinic level, we will continually engage stakeholders to discuss possibilities in the case of proof of concept to expand platform integration to include other NCDs requiring long-term management (eg, diabetes) according to local priorities. As a first step toward informing policies and the potential scale-up of our intervention in the Ugandan setting, we will engage local policy makers in qualitative interviews to discuss the wide-range potential and adaptability of our program. We plan for further engagement of these key stakeholders through dissemination workshops at the conclusion of data collection to ideate on how to further develop and grow our program.

### Limitations

With this study being a design study, the available sample size and limited generalizability owing to the specificity of patients with hypertension at 1 health facility might prove not to be sufficient to establish large-scale statements regarding intervention efficacy. Therefore, in the case of proof of concept, we encourage further randomized testing of the developed intervention with representative populations to allow for more robust interpretation and recommendations. Although the specific setting of an established dedicated NCD clinic and a patient-initiated money-saving program is one of our study’s’ key strengths, it could also prove to be a potential limitation in terms of transferability to settings with less ideal conditions. Although restricted, recent project development addressing NCDs in this region has emphasized the potential of HCD, mHealth, and community-led intervention to guide future policy decisions [68]. We will therefore refrain from making sweeping statements across settings but encourage further research on these topics in other contexts.

### Conclusions

Our study will contribute to filling a gap in the literature by investigating the design and implementation of an mHealth intervention based on an existing community-led financing scheme. We hope to spark discourse and inform future research on the potential of design research in exploring novel approaches to facilitate sustainable access to medication in resource-constrained settings. We also aim to broaden the scope of established practices for community involvement in the design and implementation of interventions by focusing on their potential for expanding end user–driven solutions already established in the field.

## Abbreviations

ACCESS: African Community Center for Social Sustainability
CHW: community health worker
CVD: cardiovascular disease
FGD: focus group discussion
HCD: human-centered design
HCP: health care provider
IDI: in-depth interview
mHealth: mobile health
NCD: noncommunicable disease
REDCap: Research Electronic Data Capture
SSA: sub-Saharan Africa
USSD: unstructured supplementary service data
WP: work package

## Data Availability

All data collected in the context of this study will be managed at the African Community Center for Social Sustainability (ACCESS) research facility in Nakaseke, Uganda. Data sharing will take place in compliance with the ACCESS data sharing policy and established procedures. Third-party researchers who are interested in the data must complete and sign data sharing agreements. Decisions on data sharing will be made on a case-by-case basis by the investigators upon reasonable request. Individuals granted database access will be expected to use the data to answer the questions clearly outlined in the data sharing agreement.
